# *Knife’s edge*: Balancing immunogenicity and reactogenicity in mRNA vaccines

**DOI:** 10.1038/s12276-023-00999-x

**Published:** 2023-07-10

**Authors:** Jisun Lee, Matthew C. Woodruff, Eui Ho Kim, Jae-Hwan Nam

**Affiliations:** 1 Department of Medical and Biological Sciences, The Catholic University of Korea, Bucheon, Gyeonggi-do 14662 Republic of Korea; 2 Department of Medicine, Division of Rheumatology, Lowance Center for Human Immunology, Emory University, Atlanta, GA USA; 3 Emory Autoimmunity Center of Excellence, Emory University, Atlanta, GA USA; 4 Viral Immunology Laboratory, Institut Pasteur Korea, Seongnam, 13488 Republic of Korea; 5 BK Plus Department of Biotechnology, The Catholic University of Korea, Bucheon, Gyeonggi-do 14662 Republic of Korea

**Keywords:** RNA vaccines, RNA vaccines

## Abstract

Since the discovery of messenger RNA (mRNA), there have been tremendous efforts to wield them in the development of therapeutics and vaccines. During the COVID-19 pandemic, two mRNA vaccines were developed and approved in record-breaking time, revolutionizing the vaccine development landscape. Although first-generation COVID-19 mRNA vaccines have demonstrated over 90% efficacy, alongside strong immunogenicity in humoral and cell-mediated immune responses, their durability has lagged compared to long-lived vaccines, such as the yellow fever vaccine. Although worldwide vaccination campaigns have saved lives estimated in the tens of millions, side effects, ranging from mild reactogenicity to rare severe diseases, have been reported. This review provides an overview and mechanistic insights into immune responses and adverse effects documented primarily for COVID-19 mRNA vaccines. Furthermore, we discuss the perspectives of this promising vaccine platform and the challenges in balancing immunogenicity and adverse effects.

## mRNA vaccines: Understanding immune responses and adverse effects

Researchers must clarify all aspects of immune responses to and side effects of mRNA vaccines so that they can be used against multiple diseases. The COVID-19 pandemic drove mRNA vaccine development, with the accelerated rollout of Pfizer/BioNTech and Moderna vaccines. A team led by Eui Ho Kim (Institut Pasteur Korea, Seongnam) and Jae-Hwan Nam (Catholic University of Korea, Bucheon) reviewed understanding of mRNA vaccine immune responses using mainly data from COVID vaccinations. The first dose of these cost-effective life-saving vaccines stimulated a robust antibody response but boosters were needed to maintain strong immunity. Recent progress in activating antigen-specific B-cells will enable diversified mRNA vaccines that protect against multiple virus strains. Side effects are often related to vaccine components. Further fine-tuning of mRNA vaccines will reduce the excessive immune responses associated with adverse reactions.

## Introduction

For more than 100 years, public vaccination campaigns have remained our most successful interdictions into the prevention of human infectious diseases. Prophylactic vaccination has been implemented to increase life expectancy and improve public health, thereby saving countless lives^1,2^. However, while vaccine technology has significantly advanced over the past century^3^, the conceptualization of an “optimal” vaccine has only grown more complicated with the rapid progress in our understanding of the cellular components associated with protective immunity. Conventional vaccines have often failed against difficult-to-target and antigenically variable viruses such as human immunodeficiency virus (HIV) and hepatitis C virus (HCV)^4^, and due to both the time required for development processes and rapid mutation of the viral genome, some traditional vaccine technologies are poorly suited for sudden outbreaks that threaten global health and security, such as the recent coronavirus disease (COVID-19) pandemic. Considering these constraints, messenger RNA (mRNA) vaccines have represented an attractive alternative to conventional vaccines due to their cell-free, rapid, and scalable development and production^5^.

mRNA vaccine technology has been extensively studied for cancer treatment owing to its ability to trigger a potent T-cell response, well-tolerated nature, and suitability for personalized design. The details of mRNA-based cancer vaccine development are comprehensively described in recent reviews^6–8^. However, it was the COVID-19 pandemic that incited interest in mRNA for medical application, leading to expedited licensure of two mRNA vaccines^9,10^, with several others at various stages of development or in clinical trials^11^. The unprecedented pace of the SARS-CoV-2 vaccine rollout was accomplishable only because of the unique properties of mRNA vaccine platforms. Tremendous advancements have been made in the efficient delivery and expression of antigenic mRNA for current COVID-19 mRNA vaccines. Modifications such as N1-methylpseudouridine, 5′ capping, and codon optimization have been used to optimize mRNA production to maximize antigen availability^12,13^. To enable efficient delivery of mRNA to the cytosol of target cells, lipid nanoparticles (LNPs) comprised of ionizable cationic lipids, cholesterol, phospholipids, and polyethylene glycol (PEG) have been used. The LNP–mRNA complex is neutral at physiological pH but becomes positively charged when the LNP is sequestered and acidified in the endosome; this process is followed by fusion with the endosomal membrane and the release of mRNA into the cytosol^13–15^. Composed solely of modified mRNA encapsulated by an engineered LNP^16^, this approach eliminates the need for the massive pathogen culturing efforts required for attenuated or killed/split vaccines, such as the polio^17^ or influenza vaccine^18^. De novo DNA template synthesis and basic molecular biology propagation allow this technology to significantly outpace even protein subunit-based approaches, resulting in nimble, cost-effective platforms capable of rapidly deploying to the front line to improve public health^19^.

The COVID-19 pandemic has allowed for a real-time assessment of the strong potential of mRNA vaccines to rapidly reshape the worldwide landscape of a deadly emerging infectious disease. First-generation SARS-CoV-2 vaccines were fully designed within a few weeks of the publication of the full-length Wuhan strain spike protein sequence^20^ and rolled into phase one clinical trials initiated within 4 months of the virus’s identification^21^. Despite the rapid pace of development and limited opportunity for design optimization, the resulting vaccines performed admirably in phase three trials with initial reported efficacies of 94–95% in preventing general illness by triggering the robust production of neutralizing antibodies and moderate T-cell responses, thus placing them among some of the most successful vaccines ever developed^22^. The rapid production and distribution of the resulting vaccines resulted in an estimated prevention of 14.4 million deaths in the first year of their availability^23^.

Despite their function as rapid countermeasures to pandemic situations, various side effects of mRNA vaccines, ranging from relatively common local reactogenicity to rare serious disease outcomes, have been reported. This review summarizes the current understanding of the balance of immune stimulation and undesired adverse events induced by mRNA vaccines and speculates future directions for developing more effective and safer vaccines using this promising vaccine technology.

### mRNA vaccine-induced immune responses and mechanism of action

**Fig. 1:**
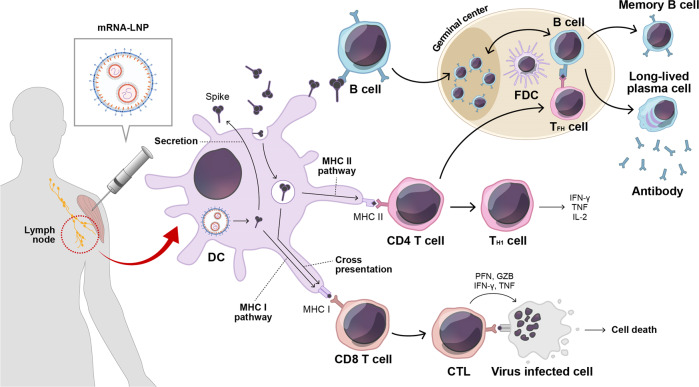
COVID-19 mRNA vaccine-induced immune responses. After mRNA vaccination, secreted spike antigens are identified by cognate B-cells and induce potent neutralizing antibody responses with a strong germinal center reaction. Dendritic cells (DCs) uptake soluble spike antigens and stimulate antigen-specific CD4 and CD8 T-cells via the MHC II and cross-presentation pathways, respectively. In addition, endogenously expressed spike proteins in DCs can activate antigen-specific CD8 T-cells through the MHC I pathway. LNP, lipid nanoparticle; FDC, follicular dendritic cell; TFH, T follicular helper cell; TH1, type 1 T helper cell; CTL, cytotoxic T lymphocyte; PFN, perforin; GZB, granzyme B; IFN-γ, interferon gamma; TNF-α, tumor necrosis factor-alpha.

Recently, approved mRNA vaccines for COVID-19 from Pfizer/BioNTech and Moderna employed similar technological paths by utilizing antigenic mRNA encoding a nucleoside-modified prefusion form of the spike antigen (S-2P) packaged in ionizable cationic LNPs for delivery. Both formulations resulted in the strong induction of protective immunity in animal models and human vaccinees^13^. The immune response triggered by COVID-19 mRNA is characterized by robust production of spike-binding and neutralizing antibodies, as well as intermediate levels of T-cell responses (Fig. 1). Notably, after the first vaccination, moderate innate immune responses, including antiviral and interferon responses, were observed, while much broader and stronger inflammatory responses, such as sharp increases in inflammatory monocytes and IFN-γ, were observed after the boost immunization^24^.

Despite clear-cut evidence of the efficacy of mRNA vaccination obtained throughout the pandemic^9,25^, challenges remain. Inefficient B-cell targeting has continued to hamper broadly neutralizing SARS-CoV-2 vaccination efforts^26^, as it has in HIV^27^ and influenza^28^. Characterization of independent pathways associated with B-cell activation and developmental biases in short-term effector, as well as long-term memory populations, has sparked interest in how differential activation of these pathways might contribute to vaccine potency^29^, targeting^30^, and reactogenicity^31^. Furthermore, the integration of robustly stimulated humoral responses with effective T-cell-mediated immunity, known to be critical in mounting primary immune responses, remains a currently unattainable goal in vaccine-induced protection, particularly at mucosal sites^32^. To address these challenges, it is crucial to fully understand the underlying mechanisms involved in immune responses to mRNA vaccination to maximize efficacy in the expanding arena of mRNA-based therapeutics.

### Antibody production and targeting

The induction of neutralizing antibodies by SARS-CoV-2 mRNA vaccines is the main correlate of protection from infection and serious COVID-19 outcomes. Despite widespread success in stimulating robust antibody titers against the SARS-CoV-2 spike protein^33^, coupled with strong neutralizing reactivity against the receptor binding domain (RBD)^34^, first-generation mRNA vaccines have been insufficient in sustaining widespread protection. Indeed, the limitations of fixed-strain vaccination against a rapidly mutating RNA virus were obvious. The emergence of the SARS-CoV-2 Alpha strain (B.1.1.7) in South Africa during efficacy testing and the resulting decrease in efficacy raised an almost immediate red flag that small modifications in RBD primary structure might hamper overall vaccine effectiveness^35^. These fears were confirmed with the emergence of the Omicron subvariants, now boasting a heavily modified RBD, which resulted in a 30-fold reduction in neutralization capacity compared to that of the Wuhan strain-targeted mRNA vaccine responses^36^.

While unfortunate, this loss of reactivity against new and emerging viral variants is not entirely unexpected. Despite significant improvements in the elicitation of antibody titers through updated vaccine platforms, most recently in mRNA-based platforms, the ability to direct in vivo responses toward intended antigen epitopes is still lacking^37^. As a result, while current mRNA vaccination platforms show a strong ability to elicit robust humoral responses, scientists largely remain passive observers in the B-cell selection processes governing epitope targeting. Reactivity against undesired but immunodominant epitopes is likely to continue to challenge the development of cross-strain protective immunity, as has been shown in previous studies investigating HIV and influenza^27,28^.

The flexibility in mRNA vaccine design, coupled with new advances in the basic science of B-cell epitope selection, offers exciting new paths forward. In particular, the identification of a new governing principle in B-cell selection, rare epitope suppression, may provide a unique opportunity alongside mRNA vaccination to generate epitope-targeted responses^38^. By diversifying antigen species within a single vaccine dose, responding B-cells are placed in competition with each other for shared pools of T-cells. Thus, mRNA species may be diversified such that epitopes derived from conserved regions, likely to provide cross-protection against multiple variants, can be emphasized over strain-specific responses. Although still in its early phases of testing, paratope-focusing has been indirectly demonstrated as a plausible method for the development of chimeric nanoparticles that incorporate multiple influenza hemagglutinin species, resulting in increased generalized cross-reactivity^39^. Multivalent approaches in mRNA vaccination are under current investigation, with the currently available bivalent SARS-CoV-2 “updated” vaccine boosters as an important proof of concept^40^.

### B-cell activation and memory development

In addition to challenges in B-cell selection and epitope targeting, response durability has become a primary concern in mRNA vaccination efforts against SARS-CoV-2^33^. While early results suggested significant and persistent antigen-specific B-cell memory formation after the initial 2-dose series, analysis of initial vaccinated patient cohorts suggested a marked drop-off in circulating antibody response with an anti-spike IgG half-life of 30 days^41^. The result was tapering vaccine efficacy^42^, ultimately leading to the proposal and recommendation of an additional booster dose to maintain anti-viral titers and host protection^43^. Although this three-dose series is not uncommon among subunit vaccines, the durability profile of first-generation mRNA vaccines did not match that of historical vaccines of similar initial efficacy, such as polio and vaccinia vaccines, where titers are known to persist for decades without the need for additional booster doses^44^.

This is, perhaps, unsurprising, given the emerging complexity of B-cell responses responsible for the generation of lifelong humoral immunity. While much focus has been placed on the traditional germinal center (GC) responses assumed to be primarily responsible for vaccination response development^45^, an important emerging feature of primary immune responses, particularly those emerging in high-inflammation environments, is an emphasis on the extrafollicular (EF) B-cell pathway^46^. Initially, identified in mouse infection modeling^47^ and human autoimmune disease^48^, the COVID-19 pandemic has validated this pathway as a prominent component of early humoral immunity that is particularly emphasized in patients with severe disease. In stark contrast with GC-focused responses, EF responses undergo less somatic hypermutation, and despite being well-selected against foreign antigens, they are less frequently identified in the persisting immune memory months or years following infection, although evaluation in the context of vaccination is needed^49^. Identification and characterization of this pathway make it clear that the initial humoral response is not sufficient to produce well-targeted, long-lasting memory responsiveness. Indeed, it has become clearer in recent years that even the production of fully functional plasma cells is not sufficient for long-term bone marrow engraftment, as further maturation of these cells appears to be both required and contingent on unknown parameters^50^.

This need for careful B-cell response tuning during vaccination has led to significant investigation into the mimicry of infectious microenvironments through vaccine adjuvant delivery^51^. It is now well established that carefully selected combinations of microbe-associated molecular patterns and danger signals can greatly impact innate immune activation and downstream humoral immunity^52–54^. However, the unique properties of mRNA vaccine platforms have somewhat upended these fields of study due to the built-in adjuvant properties of mRNA and LNPs. Although previous subunit vaccines have shown strong dependence on Toll-like receptor (TLR) signaling and cell death pathways to drive sufficient reactivity, humoral responsiveness to mRNA vaccines might not be closely associated with these factors since the MyD88 pathway is partially responsible for optimal antibody and B-cell responses^55,56^. In addition to MyD88, functional studies in mice have identified antiviral pathways, particularly those governed by the RIG-I-like receptor melanoma differentiation-associated protein 5 (MDA5), as additional mediators of developing humoral immunity^55^. As these systems are type-I interferon dependent and dsRNA responsive, it is easy to find a connection between these responses and “natural” antiviral immunity. It is noteworthy that LNP formulation with ionizable lipids can strongly induce the proinflammatory cytokine IL-6, which, in turn, contributes to potent humoral immunity^56^. However, the lack of first-generation response durability in contrast to long-persisting antiviral responses suggests that additional factors are needed. The use of self-replicating mRNA vectors, an approach available uniquely to mRNA vaccination platforms, will be of high interest in continuing to push vaccination microenvironments toward true viral infection mimicry^57^.

Despite warranted excitement about continuing to push the edge of viral infection mimicry with innovative vaccine design, it is also clear that the introduction of inflammatory signaling into vaccination design must be carefully balanced with tolerability. It is now clear that the overstimulation of the EF response pathway—a process dependent on high TLR7 signaling and associated with an IFN-γ-induced cytokine milieu—leads not only to short-lived humoral responses but also to the emergence of cross-reactive antibodies capable of targeting self-tissue^49^. Thus, it is clear that the balance between localized and carefully tuned inflammatory signaling alongside systemic reactogenicity is both highly complex and critical in the conceptualization and design of second-generation mRNA platforms and beyond.

### T-cell immunity by COVID-19 mRNA vaccines

Potent humoral immune responses, such as the production of neutralizing antibodies following vaccination, are considered to provide protective immunity against viral pathogens. However, a quality T-cell response is required for optimal vaccine efficacy both in fine-tuning B-cell activation and differentiation and in the removal of virus-infected cells^58–60^. For prophylactic COVID-19 vaccines, high titers of spike-binding neutralizing antibodies were closely associated with protective outcomes^36,55,61^. However, T-cell responses have also been linked to conferring protection against SARS-CoV-2 infection in certain contexts. For example, in convalescent rhesus macaques, the depletion of CD8 T-cells attenuated their protective immunity against subsequent challenge^62^. Moreover, uncomplicated recovery from COVID-19 in agammaglobulinemia patients or individuals receiving targeted anti-CD20 immunotherapy, as well as the preserved and durable responsiveness of T-cells to viral variants, imply that T-cell immunity plays a role in SARS-CoV-2 protection^63–65^.

In contrast to traditional vaccine technologies, including inactivated and protein subunit vaccines that rely on extracellular antigen capture, LNPs of current COVID-19 mRNA vaccines enable mRNAs to be released into the cytosol of target cells and produce spike proteins inside cells similar to that in the case of a viral infection^13^. Intracellular production of spike antigens promotes the classical loading of antigen-derived peptides on class I MHC and stimulates the activation of CD8 T-cells, while secreted antigens can be endocytosed and presented via class II MHC in antigen presenting cells and induce CD4 T-cell responses.

### Follicular helper T (T_FH_) cells and T helper 1 (T_H1_) cell preference

Among CD4 T-cell subpopulations, T_FH_ cells are specialized to support the GC reaction, where activated B-cells undergo massive proliferation and selection. Furthermore, they promote the differentiation of memory B and long-lived plasma cells and enhance antibody qualities through isotype switching and affinity maturation of B-cell receptors^66^. During the GC reaction, T_FH_ cells interact with B-cells by recognizing cognate peptides presented on class II MHCs of the B-cell surface and promoting the expansion and survival of antigen-specific B-cells by providing costimulatory molecules and cytokines, such as IL-21^66,67^. Previous evaluations of mRNA vaccines demonstrated that such vaccines could trigger potent T_FH_ cell responses displaying the characteristics of both T_H1_ and T_H2_ cells in mice and nonhuman primates^68,69^. Mechanistically, the MyD88 pathway is required for mRNA vaccine-induced potent T_FH_ response, and intriguingly, LNP also works as a built-in adjuvant to mount normal T_FH_ and GC B-cell responses^56^. COVID-19 mRNA vaccines, such as BNT162b2 and mRNA-1273, also generated efficient T_FH_ cell responses with CD40L and IL-21 expression in rhesus macaques^70,71^. Notably, the COVID-19 mRNA vaccine induced higher levels of T_FH_ cells than emulsion-adjuvanted RBD vaccines^72^.

In addition to T_FH_ cells, CD4 T-cells are effectively activated and differentiate into effector T-cells that are strongly polarized to the T_H1_ response and secrete IFN-γ, TNF-α, and IL-2 upon restimulation by spike-derived peptides. A single immunization with the COVID-19 mRNA vaccine resulted in the effective generation of T_H1_-skewed polyfunctional CD4 T-cells in mice^73^. In nonhuman primates, potent IFN-γ and minimal IL-4 production was observed following BNT162b2 and mRNA-1273 administration^70,71^. The T_H1_-biased CD4 T-cell phenotype was also confirmed in several human mRNA vaccine studies^74–77^, in line with its assumed role in response against intracellular pathogens. Interestingly, T_H2_ response polarization has been linked to vaccine-associated enhanced immunity to respiratory disease in vaccines against other respiratory viral infections^78^. The aforementioned evidence implies that COVID-19 mRNA vaccines lead to desirable T_H_ cell responses following SARS-CoV-2 infection.

### Regulation of the CD8 T-cell response

Mounting the CD8 T-cell response by mRNA vaccine platforms is another relevant aspect due to intracellular antigen expression in target cells and an abundant track record of preclinical and clinical trials in the development of mRNA-based cancer immunotherapy where cytolytic T lymphocyte (CTL) activity is crucial^6,19^. Initially, divergent evidence was reported on the CD8 T-cell responses to COVID-19 mRNA vaccines, wherein detectable spike-specific CD8 T-cell responses were observed in BNT162b2-vaccinated rhesus macaques and humans. However, low CD8 T-cell responses were observed following mRNA-1273 vaccination of rhesus macaques and humans^70,71^. Nevertheless, more recent studies directly compared two approved mRNA vaccines and demonstrated similar induction of spike-specific CD8 T-cells in humans^65,79^. Notably, a mechanistic study in a mouse model revealed that the BNT162b2-induced CD8 T-cell response was conveyed by classical MHC I presentation, at least in part, as the CD8 T-cell response was not completely abrogated in cross-presentation-deficient Batf3 knockout mice^55^. Importantly, one of the cytosolic RNA-recognizing receptors, MDA5, and subsequent type I IFN signaling were required for the spike-specific CTL response following BNT162b2 vaccination^55^.

### Adverse events of mRNA vaccines

Various vaccines against SARS-CoV-2 have been developed, and their efficacies have been compared; the effectiveness of mRNA vaccines is superior to that of those derived from previously existing technologies. However, as the effectiveness increases, adverse effects need to be carefully evaluated for the safer use and advancement of current vaccines. Recently, natural killer (NK)/monocyte subsets, dendritic cell (DC) subsets, and NK T-like cells have been shown to be involved in both mRNA vaccine effects (increasing neutralizing antibody titer) and side effects^80^. In addition, these cells are related to an increase in IFN-γ-inducible chemokines. These results suggest that mRNA-induced high vaccine efficacy can be closely related to several side effects and that maintaining an appropriate balance of the two opposing responses is paramount for developing a suitable vaccine. Here, we summarized the mRNA vaccine-induced side effects and their underlying mechanisms.

### Local and systemic reactogenicity

In early clinical trials of COVID-19 mRNA vaccines, local side effects and symptoms, such as swelling, redness, pain, and heat, were more common in vaccine patients than in those receiving the placebo. Within one week of vaccination, one of the most common local reactions was pain at the injection site^81,82^. Systemic side effects, such as fatigue, headache, fever, myalgia, and arthralgia, were also more common following mRNA vaccine administration compared to those receiving the placebo. Additionally, it was reported that systemic side effects were more frequently observed in young vaccine recipients (16‒55 years of age) than in their older counterparts (>55 years of age). The higher systemic reactogenicity may imply that the immune responses are more robust in the younger population than in the older population. In comparison to the first dose, the second vaccine dose was associated with more severe side effects, such as fatigue and headache^9,10^.

### Serious adverse events

A recent report showed that although the results were preliminary and had several limitations, 0.125% of mRNA-vaccinated individuals showed severe adverse effects, including acute myocardial infarction, Bell’s palsy, cerebral venous sinus thrombosis, Guillain–Barré syndrome, myocarditis/pericarditis (mostly at younger ages), pulmonary embolism, stroke, thrombosis with thrombocytopenia syndrome, lymphadenopathy, appendicitis, herpes zoster reactivation, neurological complications, and autoimmunity^83^.

**Fig. 2:**
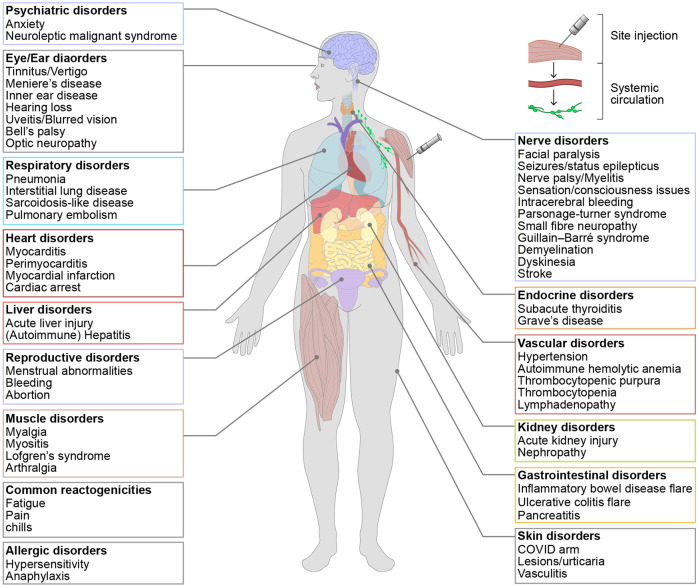
Adverse events following mRNA vaccination against COVID-19. Adverse events following mRNA vaccination against COVID-19 were sorted and summarized by organs from the head to the feet of a human body.

Anaphylaxis is a severe adverse reaction that requires immediate medical treatment. According to a recent report, approximately 1:200,000 recipients experienced anaphylaxis as a result of the Pfizer/BioNTech vaccine^10^. More recently, the incidence of anaphylaxis was estimated at 2–11 cases per million after receiving the Moderna or Pfizer/BioNTech vaccines^84^. The rate for most vaccines is less than one per million doses. Although PEG has been considered a potential causative agent for anaphylaxis, the causative antigen and associated mechanisms remain under investigation^81,85,86^. Myocarditis and pericarditis are also reported serious adverse effects of mRNA vaccination, although the rate is extremely low (0.005%)^87^. A summary of the common mild and severe adverse effects and potential complications is shown in Fig. 2.

### Risk factors and cytotoxic mechanisms associated with mRNA vaccines

mRNA vaccine safety is, at least in part, derived from the tolerability of the lipid and mRNA components included in the formulation. Lipids have been previously shown to induce host immune responses following vaccine administration, and reactivity to repeated administrations of PEG-based nanoparticles through complement-mediated mechanisms may negatively affect safety and efficacy profiles^88^. Another safety concern is the immunogenicity of in vitro transcript (IVT) mRNA, despite the potential advantage of stimulating cellular and humoral immunity for proper vaccination^89–91^.

**Fig. 3:**
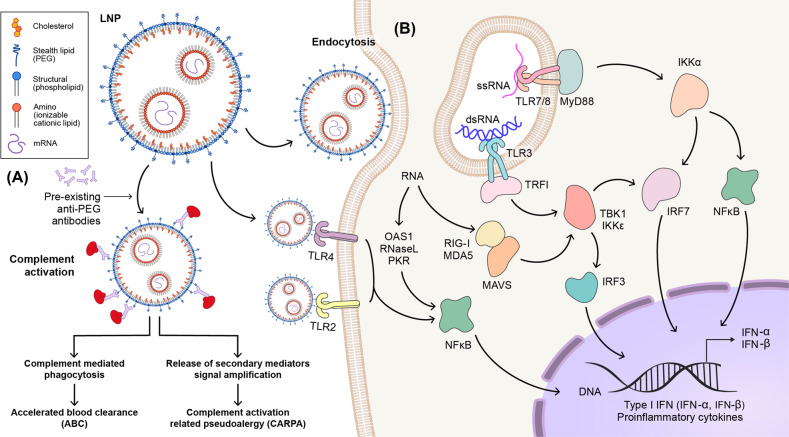
Risk factors and the cytotoxic mechanisms of mRNA vaccines. **A** LNP-induced immune activation. Schematic structure of mRNA encapsulated into LNP formulations composed of an ionizable cationic lipid, helper lipid, cholesterol, and PEG. LNPs can induce immune activation by stimulating Toll-like receptor (TLR) 2 and TLR4 and leading to NF-kB activation and cytokine secretion. Preexisting anti-PEG antibodies can lead to complement activation and subsequently complement-mediated phenomena, such as ABC or CARPA. **B** LNP-encapsulated mRNA is taken up by immune cells through endocytosis. In endosomes, TLR7/8 and TLR3 recognize ssRNA and dsRNA, respectively, and the receptors activate MyD88 and TLR3 in Toll-interleukin-1 domain-containing adapter-inducing interferon (TRIF). Eventually, the related signaling cascades transduce to the nucleus where type I IFN and pro-inflammatory cytokine production is promoted by transcription factors (NF-kB, IRF3, and IRF7). Endosomal escape is used to transport small amounts of mRNA and IVT reaction byproducts to the cytoplasm. The RNAs are recognized by RIG-I and MDA5 and then both signaling pathways activate the transcription factors for inflammatory gene expression.

As mRNA vaccines use single-stranded RNA, it was expected that the immune response would be induced through TLR7/8. However, recent reports have shown that mRNA vaccines lead to the production of type I interferon (IFN-I) via MDA5 and not TLR7 and proinflammatory cytokines, such as IL-1β and IL-6^55,56^. These induced IFN-I and proinflammatory cytokines have the benefit of stimulating immune responses and improving vaccine efficacy, whereas they may also induce immunological adverse effects. Meanwhile, the complement system is activated when PEG, one of the LNP components, interacts with anti-PEG antibodies, which are already present in the body. However, it is merely postulated that complement-mediated phagocytosis may elicit adverse effects, such as rapid blood clearance and complement activation-related pseudoallergy (CARPA)^92^. In this section, we review the potential risk factors of mRNA vaccines consisting of LNPs and mRNA. A selection of adverse events with proposed associated mechanisms is delineated in Fig. 3.

### LNP

#### Immunogenicity of LNP

The physical and chemical properties of LNP, such as its shape and charge, affect its interaction with the immune system. A previous study showed that LNP, as a built-in adjuvant, elicits potent antigen-specific CD4^+^ T_FH_ cell and GC B-cell responses via induction of IL-6 production, independent of MAVS-mediated RNA-sensing pathways^93^. Strong immunogenicity is often responsible for reactogenicity (even toxicity) that causes local and systemic inflammation. Therefore, to ensure the efficacy and safety of mRNA vaccines, it is important to comprehend the molecular mechanism(s) by which adjuvants, such as lipid components, prompt the immune system^94^. Several recent studies have demonstrated that LNPs with ionizable lipids trigger proinflammatory cytokines, including IL-1β and IL-6, and subsequently, antibody and T-cell responses^56,95^. Moreover, the complement system and TLRs may participate in the activation of the innate immune system^90^.

#### Components and toxicity of LNP

LNP is a multicomponent lipid system that is comprised of cationic/ionizable lipids, cholesterol, helper lipids, and PEG-lipids, among others. Several studies have thus far investigated the structure-activity relationship of LNPs and the mechanisms through which LNPs interact with the immune system based on their particle size, charge, hydrophobicity, component molar fraction, and surface chemistry^96,97^. LNPs can cause various immune effects in vivo, including immune cell activation, inflammation, adaptive immune response, and complement activation, as well as CARPA, depending on their properties and method of delivery^98–100^.

#### Cationic/ionizable lipids

Cationic lipids, such as 1,2-di-O-octadecenyl-3-trimethylammonium-propane (DOTMA)^101^ or 1,2-dioleoyl-3-trimethylammonium-propane (DOTAP), were used to facilitate mRNA encapsulation in the earliest liposomal delivery systems^102^. Cationic-based mRNA delivery systems have been proven to be related to innate immune responses in vitro and in vivo^103,104^. Cationic lipid nanocarriers induce the dimerization and activation of TLR2 and TLR4 proteins on antigen-presenting cells, such as DCs and macrophages, owing to their small size and electrical properties^105–107^. Consequently, the LNP–TLR complex triggers the secretion of various proinflammatory cytokines and chemokines through similar signaling pathways^108^ and promotes the formation of the NLRP3 inflammasome^98,105^. Therefore, based on safety concerns, cationic lipids have not been considered suitable materials for developing current mRNA vaccines. However, there have been attempts to develop ionizable lipids that are positively charged by the protonation of free amines at acidic pH, allowing for RNA complexation in an acidic buffer while remaining neutral at physiological pH. This ability of ionizable lipids plays a role in endosomal escape and RNA cytosolic delivery by forming destabilizing non‐bilayer structures, with a lowering of the pH^103^. Thus, pH-sensitive protonation or ionization properties of lipid materials in physiological and endosomal conditions are required for safe and effective LNP architecture in clinical use with systemic administration. These pH-sensitive ionizable lipids have little or no amphiphilicity at physiological pH but exhibit high amphiphilicity at endosomal pH, significantly reducing cytotoxicity and side effects in vivo. In addition, the destabilization of pH-sensitive amphiphilic cell membranes can be controlled by the gradient of LNP concentrations^109^. Furthermore, the pH-sensitive ionized lipids likely minimize cellular toxicity and adverse reactions through stable LNP formation and efficient intracellular delivery of nucleic acids at low N/P ratios, which can also be improved by surface modification of LNPs using biocompatible polymers^110,111^. Thus far, three ionizable cationic lipids, ALC-0315, SM-102, and DLin-MC3-DMA (MC3), have been approved for clinical use^112^. For the COVID-19 vaccines, Moderna used SM-102, and Pfizer/BioNTech employed ALC-0315 licensed from Acuitas^113^; these lipids are remarkably similar in structure. Recently, in a comparative study of ionizable cationic lipids for RNA therapy, ALC-0135 LNPs showed much higher siRNA knockdown efficiencies than MC-3 LNPs with markedly lower toxicities^112^. Taken together, these data reveal that ionizable cationic lipids are crucial components of RNA therapeutics required for maximal efficacy with limited toxicity.

#### PEG

PEGylation of LNPs is widely utilized to render stability and increase the plasma half-life. Currently, more than fifteen PEGylated drugs, including Doxil®, Onpattro®, Pfizer/BioNTech COVID-19 vaccines (COMIRNATY®), and MODERNA COVID-19 VACCINE®, have full approval or emergency authorizations worldwide^114^. Although PEG-based nanoparticles display low reactogenicity profiles^115^, the generation of anti-PEG antibodies is a potential issue that can be overcome with the repeated administration of PEGylated pharmaceuticals^92,99^.

For the first time in 1983, the immunogenicity of PEG was discovered through an assessment of the production of anti-PEG antibodies by injecting PEGylated proteins into rabbits^116^. The widespread use of PEG molecules in various industries has increased the percentage of positive healthy volunteers with anti-PEG antibodies, from approximately 0.2% in 1984 to 40% in 2016^98^^,^^99^. The preexisting or de novo anti-PEG antibodies activate the complement system. As recently indicated in the case of the two LNP–mRNA vaccines for COVID-19, mRNA-1273 (Moderna) and BNT162b2 (Pfizer/BioNTech), PEGylated LNPs induced anti-PEG antibodies with the activation of the complement system; thus, their uses possibly put the vaccine recipients at risk of allergic reactions^117^.

Activation of the complement system can accelerate blood clearance (ABC) and significantly reduce the therapeutic efficacy of LNP–mRNA vaccines^99,118^. Additionally, anti-PEG antibodies have been linked to pseudo-allergic reactions in patients who received PEGylated drugs through intravenous administration, potentially leading to anaphylactic shock and death^100,119^. These hypersensitivity reactions are considered to occur through CARPA activation^92,119^, which is the primary mechanism of infusion reactions, including pseudo-anaphylactic shock^98^. Therefore, developing PEG-free delivery systems is necessary for future mRNA vaccine development. In this regard, exploring the properties of various materials, such as synthetic, natural, and zwitterionic polymers, is suggested for the development of more effective and safer mRNA delivery systems. These materials have been recognized for their potential to offer innovative and promising medicinal applications compared to PEG. However, whether they provide therapeutic benefits without eliciting pathological immune responses or side effects is uncertain. Therefore, guidelines should be established for screening and accurately determining the degree of immunogenicity of potential candidates^120^.

### Toxicity of the mRNA platform

Regarding mRNA biomolecules, exogenous RNA can expose individuals to endothelial damage, intercellular junction relaxation, edema, increased viscosity, hypercoagulation, and thromboembolic events due to the immunogenicity of the exported RNA^19^. To abate exogenous RNA-induced immunogenicity, two technical approaches are commonly available. First, chemical modification of IVT mRNA molecules with pseudouridine (ψ) or N1-methylpseudouridine (m1ψ) allows the mRNA to avoid innate immune sensing that occurs through the interaction of exogenous mRNA with TLR7^121,122^. m1Ψ-modified mRNA has been shown to exhibit less cytotoxicity and immunogenicity than wild-type mRNA. The interference of intracellular innate immune signaling provides advantages for the replacement of conventional protein therapy with the mRNA platform. Second, chromatographic purification can attenuate immune reactions and enhance translational efficiency to eliminate double-stranded RNAs, such as analogs of the viral genome, during IVT mRNA preparations^123^. However, it may require optimization for mRNA vaccination, as inflammatory cytokines have been shown to boost adaptive immune responses. Therefore, further fine-tuning of the levels of base modifications may be required for mRNA vaccination to balance exogenous mRNA translation and innate immune stimulation.

Modified nucleosides, which have previously been applied in antiviral therapy, cannot be incorporated into the RNAs of natural organisms natively^124^. Theoretically, chemically modified unnatural molecules can improve the properties of natural mRNAs. However, intensive investigation of their safety is needed before therapeutic inclusion because the administration of unnaturally modified nucleoside molecules into human individuals has previously caused mitochondrial toxicity, liver failure, and even death during clinical trials^125^. Therefore, the use of base modifications naturally found in RNAs might be a safer strategy for therapeutic applications^126^.

### Conclusions and future directions

mRNA vaccines gained considerable attention during the COVID-19 pandemic due to their great advantages associated with rapid manufacture, reasonable vaccine efficacy, acceptable tolerability, and broad applications relevant to therapeutic fields, including oncology and enzyme replacements, as well as prophylaxis. Their applicability can be further improved by preventing related adverse effects and reducing risk factors associated with the use of modified mRNA and LNP, as well as overcoming unstable durability and potentiating CD8 T-cell responses. Despite these challenges, the flexibility and cost-effectiveness of mRNA platforms offer unique strategies to further improve and solve long-standing challenges associated with vaccine design^127^. Due to their widespread current use^128^ and likely trajectory as a platform of choice in a variety of human vaccination efforts^19^, it is important to evaluate potential avenues for investigating both their ability to overcome these challenges and their potential impact on reactogenicity and tolerability in humans. In addition, it is important to simultaneously identify potential disease targets, as the mRNA vaccine platform may not be a universal solution. However, considering the immense potential of mRNA vaccines in mitigating human disease, significant efforts in clarifying all aspects of this exciting new technology are both warranted and needed.
